# Diet quality of Chilean schoolchildren: How is it linked to adherence to dietary guidelines?

**DOI:** 10.1371/journal.pone.0318206

**Published:** 2025-02-06

**Authors:** Anna Christina Pinheiro Fernandes, Jacqueline Araneda Flores, Daiana Quintiliano Scarpelli Dourado, Tito Pizarro Quevedo, Maria Rita Marques de Oliveira

**Affiliations:** 1 Carrera de Nutrición y Dietética, Facultad de Medicina Clínica Alemana, Universidad del Desarrollo, Las Condes, Santiago, Chile; 2 Faculty of Health and Food Sciences, University of Bío-Bío, Chillán, Chile; 3 Faculty of Medical Sciences, University of Santiago de Chile, Santiago, Chile; 4 Institute of Biosciences, São Paulo State University (UNESP), Rua Prof. Dr. Antônio Celso Wagner Zanin, Botucatu, SP, Brazil; Lusofona University of Humanities and Technologies: Universidade Lusofona de Humanidades e Tecnologias, PORTUGAL

## Abstract

**Introduction:**

In Chile, the prevalence of overweight among schoolchildren over the age of 5 is approximately 50%, one of the highest in the Americas region. This is largely influenced by the presence of inadequate food environments, characterized by limited access to healthy foods and a high availability of highly processed foods (HPF). This study analyzes food consumption in Chilean schoolchildren and the association of this with the Chilean Dietary Guidelines Based on Foods (DGBF) and the HPF consumption.

**Material and methods:**

The sample (1,094 schoolchildren) was obtained from the baseline of the FONDEF IT18I0016 project, in Santiago-Chile. Anthropometry and socioeconomic data were measured. Dietary intake was assessed using a validated semi-quantitative frequency survey featuring images of food groups aligned with DGBF, as well as HPF. All data were collected by trained professional nutritionists. Associations between dependent and independent variables, and potential confounding factors were calculated using logistic regression models with stepwise backward/forward. A p < 0.05 was accepted as significant, using Stata 16.0.

**Results:**

Most of the students (54.1%) were with malnutrition by excess and 20.0% exhibited abdominal obesity. Twenty percent of the students met the recommended intake for DGBF (fruits, vegetables, fish, milk and dairy products, legumes, water) and less than 5% did not consume any HPF. Consuming some sub-groups of HPF in moderate quantities (either no consumption or occasional consumption) increases the likelihood of meeting DGBF: candies and sweets (OR: 0.72; p = 0.04), pies with or without filling (OR: 0.63; p = 0.01), and salted snacks (OR: 0.67; p = 0.02).

**Conclusion:**

Our results contribute to reaffirm the need to enhance healthy food consumption among schoolchildren and to discourage the intake of HPF, particularly focusing on specific sub-groups of HPF that may increase the risk of displacing the consumption of food groups recommended by the DGBF.

## Introduction

Chile presents a high prevalence of obesity both in children and adults. Over 70% of the population aged 15 years old and older has malnutrition by excess, and more than 50% of children in 1^st^ grade (seven/eight years old) have the same condition. Excess weight in Chile is influenced by socioeconomic determinants of health, with the most affected groups being individuals with low education level, those living in vulnerable territories, and families where the head of the household is a woman [1–3]. The low consumption of healthy foods (fruits, vegetables, fish, legumes, dairy) and the high consumption of processed and highly processed foods (HPF) are strongly related to social and economic determinants of health [4, 5].

Obesity and other chronic diseases related to food consumption are among the most significant public health problems, affecting most countries in the Americas region and impacting individuals at all stages of the life cycle. The factors contributing to obesity are similar across different countries and are generally influenced by the presence of inadequate food environments. These environments are characterized by a low availability of healthy foods and a high availability of unhealthy foods, particularly HPF [5]. Strategies that involve the implementation of structural measures alongside nutritional education are considered essential for addressing and mitigating the issue.

Food and nutrition education in Chile is grounded in the Chilean Dietary Guidelines Based on Foods (DGBF), developed by the Ministry of Health for the general population [6], as well as an adapted version for children and adolescents aged zero to eighteen [7]. These guidelines outline appropriate food consumption based on age groups, and the recommendations have been utilized to analyze compliance with dietary consumption as defined by the DGBF in various national surveys [8, 9].

According to the National Food Consumption Survey, in Chile, only 41.0% (36.0%-48.1%) of children aged 6–13 years old consume the recommended amount of fruits and vegetables (5 portions/day); 37.8% (25.2%-40.3%) consume the dairy recommendation (3 portions/day); 28.3% (22.2%-34.3%) consume the legumes recommendation (2 portions/week); and only 16.3% (12.4%-20.2%) consume the fish recommendation (2 portions/week) [8]. Snack consumption among schoolchildren in Chile provides over a quarter of their daily energy intake with high consumption of foods high in energy, saturated fat, sodium and/or total sugars [8, 10].

The consumption of HPF is associated with an increased risk of chronic diseases, particularly obesity, regardless of the food classification used. Food classification is a complex process, and the most used classifications in the literature are NOVA (four categories), International Food Information Council (IFIC) (five categories), and University of North Carolina (UNC) (seven categories) [11–13].

The proportion of food consumption by NOVA, which classifies food groups by the degree of processing, varies according to each country. In Chile, ultra-processed foods contribute 38.0% and 34.0% of the total calories for children aged 6–11 years old and 12–19 years old, respectively. A similar distribution is observed in Mexican children (34.0% and 36.0%). Unprocessed food contributes 32.0% of total calorie intake in Chile and 51.0% and 49.0% in Mexican children aged 6–11 years old and 12–19 years old, respectively [14].

In the region, some countries are working to implement structural regulations to establish healthy food environments and improve the quality of diets, particularly for schoolchildren [15]. Since 2016, Chile has implemented the "Food Law" which introduced food advertising labels on packaged foods high in critical nutrients. The evaluation of this regulation indicates that the industry reduced the content of critical nutrients (primarily sugars) to comply with the regulation [13]. As a result, the total sugar consumption by schoolchildren decreased by 4.5 percentage points (pp) (-8.0, -0.9) in 2018 and 11.8 pp (-15.4, -8.3) in 2019 [16]. However, a negative consequence observed after the enforcement of the law is the increased consumption of non-nutritive sweeteners [17].

Considering the risk of chronic diseases associated with the consumption of HPF, we hypothesize that this food group is significantly consumed by children and adolescents in Chile. Therefore, it is essential to analyze how this consumption interferes in the probability of complying with the food consumption recommendations of the DGBF. This study examines the association between Chilean children’s dietary habits and adherence to the Chilean DGBF, focusing on the impact of HPF.

## Material and methods

The sample was obtained from the baseline of the FONDEF IT18I0016 project, conducted between October 2019 and March 2020. A total of 1,340 schoolchildren were included in the study. During the data cleaning process, schoolchildren lacking relevant information for these analyses were excluded. A total of 242 schoolchildren had no weight data, 246 no height data, 245 had no waist circumference data, and 236 did not provide birthdate information, making it impossible to calculate their age.

Finally, the study included 1,094 children evaluated from seven municipal schools located in the southern area of Santiago, Chile. These schools are situated in the communes of El Bosque, La Granja, Lo Espejo, Pedro Aguirre Cerda, San Joaquin, and San Ramon. Data registration and storage were managed using REDCap 14.1.2 electronic data capture tool hosted at the Universidad del Desarrollo, Chile [18].

The study design adopted a cross-sectional approach, with school selection guided by local stakeholders from Municipal Education Departments. This process considered several factors: the School Vulnerability Index (IVE), assessed annually by the Ministry of Education; the extent of ongoing interventions at each school related to healthy eating and physical activity [19]; and the willingness of school principals to participate in the intervention project for a specified 2-year period. Inclusion criteria for student selection considered: enrollment in the chosen school for over a year, residence in the school’s neighborhood, age between 8 and 13 years old (from second to fourth grade), guardian’s informed consent, and the child’s informed assent. Exclusion criteria encompassed the use of restrictive diets due to diagnosed chronic conditions such as diabetes, celiac disease, or renal pathology.

The sample for the baseline of the FONDEF IT18I0016 project was selected using proportional stratified probability sampling by allocation. The population was defined as the total number of students enrolled in primary education across each municipality, based on data from the Communal Statistical Reports of the six participating municipalities [20].

Variance in grip strength among students, as reported by Garcia-Hermoso et al. [21] was considered, aiming for a precision of 0.1 kg at a 95% confidence level. This calculation determined a required sample size of 1,936 students, with an anticipated 20% attrition rate. All data were collected in-person by trained and standardized nutritionists and physical education teachers.

Sociodemographic and dietary information regarding the children was gathered using specially designed questionnaires as part of the FONDEF IT18I0016 project. All instruments utilized are validated for Chilean population. The protocol was approved by the Ethics Committee of the Universidad de Santiago de Chile (record number 187/2019). Written informed consent from the parents/guardians was obtained in person during the regular school meeting, prior to any contact with the children. Only the children whose parents/guardians had signed the consent form were recruited. For these children, informed assent was read aloud by the interviewer and then signed by the children. All personal data were anonymized and coded before analysis.

To assess the dietary intake of the students, the survey developed by Lera et al. 2015 [22], validated for Chilean schoolchildren was employed. This instrument uses a semi-quantitative frequency survey featuring images of food groups aligned with Chilean Dietary Guidelines Based on Foods [7], for the identification and quantification of food groups consumption and the dietary intake data were collected by trained professional nutritionists. To evaluate the degree of compliance with the dietary guidelines, the following servings were considered adequate: 3 servings of dairy per day, 2 servings of fish and legumes per week, 3 servings of vegetables and 2 servings of fruits per day, 1 serving of bread per day (6–10 years) /2servings per day (11–18 years) and 6 to 8 glasses of water per day. The dependent variable considers the compliance of three or more DGBF [8].

Although the consumption of HPF is not recommended, in the group studied, non-consumption reaches very low proportions; consequently, it was necessary to adjust the food consumption recommendations on the statistical analysis. Therefore, for the analysis of this item, criteria were constructed to define "acceptable consumption": sandwiches, pizzas and other items (junk food) = 1 portion or less per week; processed juices and sugary sodas = 1 serving (glass 200ml) or less per day; candies, sweets, etc. = 1 serving or less per week; salted snacks = 1 serving or less per week; pies with or without filling = 1 serving or less per week.

The nutritional status of the children was assessed by measuring weight and height to calculate the age-specific body mass index (BMI) and classify nutritional diagnosis according to guidelines from the Ministry of Health of Chile [23], being considered as malnutrition by excess those classified as overweight, obesity and severe obesity. Weight was measured using an electronic scale (Seca), and height was measured using the portable Seca 213 stadiometer. Abdominal obesity was determined using waist circumference measured with a non-extendable tape measure and classified by percentiles by age (> p 90) [23]. All anthropometric measurements were performed in triplicate, and the mean value was recorded as the final measurement.

The normality of the distribution of quantitative variables was assessed using the Kolmogorov-Smirnov test. Descriptive statistical analyses were then conducted, including measures of central tendency and dispersion based on variable distribution. For categorical variables, absolute and relative frequency analyses were used. Statistical differences were evaluated using the Chi-square test. To explore associations between dependent (compliance of three or more dietary guidelines) and independent variables, as well as potential confounding factors, logistic regression models were employed with stepwise backward and forward methods. Statistical significance was set at p < 0.05. All analyses were performed using Stata version 16.1 software.

## Results

The sample consisted of 1,094 students, comprising 55.8% males and 44.2% females. More than half of the students (54.1%) were classified as malnutrition by excess, with no significant differences observed by gender or grade level. Approximately 20.0% of the students exhibited abdominal obesity as determined by waist circumference, with prevalence consistent across all grades. Stunting affected only 1.4% of the students, with no gender or grade disparities (Table 1).

**Table 1:** Characteristics of children enrolled in 7 schools in the southern area of Santiago according to grade level, 2019 (n = 1,094).

Variable	Total sample	2^nd^ grade	3^rd^ grade	4^th^ grade	p-value
Continuous Variables, Mean± SD					
Age (Years)	9.1±1.3 (8.2–12.7)	9 ± 1.4	9.1 ± 1.3	9.1 ± 1.5	<0.001
Waist Circumference (cm)	67.0±10.7 (59–72.9)	66.8±10.4 (58.3–72.7)	67.6±10.9	66.6±10.9	<0.001
Categorical Variables, n (%)					
Gender, n (%)					
Male	610 (55.8)	233 (58.1)	166 (53.5)	211 (55.1)	0.454
Female	484 (44.2)	168 (41.9)	144 (46.4)	172 (44.9)	
Nutritional Status (BMI/age), n (%)					
Undernourished	11 (1.0)	5 (1.3)	4 (1.3)	2 (0.5)	0.603
Risk of Undernutrition	33 (3.0)	12 (1.3)	7 (2.3)	14 (3.7)	
Normal	456 (41.9)	170 (42.8)	124 (40.0)	162 (42.4)	
Overweight	286 (26.7)	98 (24.7)	77 (24.8)	111 (29.1)	
Obesity	202 (18.5)	77 (19.4)	64 (20.6)	61 (16.0)	
Severe obesity	101 (9.3)	35 (8.8)	34 (11.0)	32 (8.4)	
Nutritional Status (WC), n (%)					
Normal	581 (53.6)	216 (54.5)	159 (51.5)	206 (54.3)	0.504
Risk of abdominal obesity	274 (25.3)	101 (25.5)	87 (28.2)	86 (22.7)	
Abdominal obesity	229 (21.1)	79 (19.9)	63 (20.4)	97 (23.0)	

BMI: Body Mass Index; WC: Waist Circumference.

Analysis of adherence to DGBF reveals that around 20.0% of students meet the recommended intake for prioritized food groups in this study (fruits, vegetables, fish, milk and dairy products, legumes, water), with no variations observed by group or gender. Specific percentages for adequate consumption are reported as follows: water 21.4%, fruits 28.4%, fish 29.2%, legumes 35.4%, and milk and dairy products 35.8%. Only 9.0% of children consume the recommended daily portions of vegetables (Table 2).

**Table 2:** Food consumption profile based on Chilean Dietary Guidelines Based on Foods, children enrolled (n = 1,094) in seven schools in southern area of Santiago, Chile, 2019.

Variable	Total sample	2^nd^ grade	3^rd^ grade	4^th^ grade	p-value
Water consumption					
Less than recommended, n (%)	778 (72.0)	278 (69.3)	226 (72.9)	284 (74.1)	0.323
Adequate, n (%)	234 (21.4)	91 (22.7)	62 (20.0)	81 (21.1)	
Another quantity, n (%)	72 (6.6)	32 (8.0)	22 (7.1)	18 (4.7)	
Vegetables intake					
Less than recommended, n (%)	937 (85.6)	342 (85.3)	266 (85.8)	329 (85.9)	0.713
Adequate, n (%)	99 (9.0)	36 (9.0)	25 (8.1)	38 (9.9)	
Another quantity, n (%)	58 (5.4)	23 (5.4)	19 (6.1)	16 (4.2)	
Fruit intake					
Less than recommended, n (%)	235 (21.5)	87 (21.7)	66 (21.3)	82 (21.4)	0.431
Adequate, n (%)	311 (28.4)	116 (28.4)	98 (31.6)	97 (25.3)	
Another quantity, n (%)	548 (50.1)	198 (49.4)	146 (47.1)	204 (53.3)	
Fish intake					
Less than recommended, n (%)	758 (69.2)	279 (69.6)	205 (66.1)	274 (71.5)	0.465
Adequate, n (%)	320 (29.2)	118 (29.4)	100 (32.3)	102 (26.6)	
Another quantity, n (%)	16 (1.5)	4 (1.0)	5 (1.6)	7 (1.8)	
Legumes intake					
Less than recommended, n (%)	685 (62.7)	260 (64.8)	196 (63.2)	230 (60.0)	0.564
Adequate, n (%)	384 (35.4)	135 (33.7)	109 (35.2)	143 (37.3)	
Another quantity, n (%)	21 (1.9)	6 (1.5)	5 (1.6)	10 (2.6)	
Dairy intake					
Less than recommended, n (%)	643 (58.8)	245 (61.1)	189 (60.1)	209 (54.6)	0.306
Adequate, n (%)	392 (35.8)	136 (33.9)	107 (34.5)	149 (38.9	
Another quantity, n (%)	59 (5.4)	20 (5.0)	14 (4.5)	25 (6.5)	
Global Dietary Guidelines Adherence					
Less than recommended, n (%)	224 (20.5)	78 (19.4)	60 (19.3)	86 (22.4)	0.372
Adequate, n (%)	620 (56.6)	241 (60.1)	174 (56.1)	205 (53.5)	
Another quantity, n (%)	250 (22.8)	82 (20.4)	76 (24.5)	92 (24.0)	

Bread consumption exceeds the recommended daily intake (1 portion) for most children aged 6–10 years old (82.2%). Among children aged 11 years old and older (recommended intake: 2–4 portions daily), adequacy reaches 64.9%, with third-grade girls showing the highest adequacy (72.9%; p = 0.034).

Analysis of HPF consumption indicates that less than 5% of students do not consume this food group, while approximately 70% of students consume "junk food" items, such as sandwiches and pizza, at acceptable levels. Similar proportions are observed for sweet and savory cookies and pies. However, industrialized juices and sodas exhibit the highest levels of inadequate consumption at 87.0%, followed by candies and sweets at 35.6% (Table 3).

**Table 3:** Consumption of highly processed foods from children enrolled (n = 1,094) in seven schools in southern area of Santiago, Chile, 2019.

Variable	Total	2^nd^ grade	3^rd^ grade	4^th^ grade	Chi^2^; p
Sandwiches, pizzas, and other items (junk food)					
Exceeding the acceptable amount, n (%)	211 (19.3)	81 (20.2)	64 (20.6)	66 (17.2)	5.6029; 0.231
Acceptable, n (%)	859 (78.5)	314 (78.3)	235 (75.8)	310 (80.9)	
Another quantity, n (%)	24 (2.2)	6 (1.5)	11 (3.5)	7 (1.8)	
Processed juices and sugary sodas					
Exceeding the acceptable amount, n (%)	952 (87.0)	352 (87.8)	268 (86.4)	332 (86.7)	2. 0265; 0.731
Acceptable, n (%)	114 (10.4)	41 (10.2)	35 (11.3)	38 (9.9)	
Another quantity, n (%)	28 (2.6)	8 (2.0)	7 (2.3)	13 (3.4)	
Candies and sweets					
Exceeding the acceptable amount, n (%)	390 (35.6)	135 (33.7)	108 (34.8)	147 (38.4)	4.0463; 0.400
Acceptable, n (%)	637 (58.2)	246 (61.3)	180 (58.1)	211 (35.1)	
Another quantity, n (%)	67 (6.1)	20 (5.0)	22 (71)	25 (6.5)	
Pies with or without filling					
Exceeding the acceptable amount, n (%)	268 (24.5)	99 (24.7)	80 (25.8)	89 (23.2)	1.6600; 0.798
Acceptable, n (%)	801 (73.2)	295 (73.6)	223 (71.9)	283 (73.9)	
Another quantity, n (%)	25 (2.3)	7 (1.7)	7 (2.3)	11 (2.9)	
Salted snacks (French fries, Puffs Cheese Flavored Snacks, etc)					
Exceeding the acceptable amount, n (%)	301 (27.5)	106 (26.4)	82 (26.5)	113 (29.5)	5.6757; 0.225
Acceptable, n (%)	762 (69.6)	289 (72.1)	216 (69.7)	257 (67.1)	
Another quantity, n (%)	31 (2.8)	6 (1.5)	12 (3.9)	13 (3.4)	

Most children regularly have breakfast (94.0%), either at home (40.9%) or at school (22.9%), with 32.0% eating breakfast both at home and at school. The majority of them (85.6%) consume a morning snack, with 43.6% bringing it from home rather than buying it at school. Forty percent of children choose fruit for their snack, with over half of second-grade girls (50.6%) selecting fruit (Chi²: 8.99; p = 0.003). A similar trend is observed among fourth-grade girls, who also show higher fruit consumption (Chi²: 5.39; p = 0.020). Only a small percentage (6.9%) of students mention eating vegetables, while milk and dairy products make up for 39.5% of home-packed morning snacks.

Regarding HPF, savory snacks are included in 15.4% of morning snacks, while sweet cookies are more common (43.9%). Sugary juices and sodas make up for 36.3% of the beverages, and unsweetened juices and sodas account for 13.5%. Water is present in 19.5% of home-packed morning snacks, with a higher prevalence among second-grade students (23.2%; Chi²: 6.79; p = 0.033).

Filled bread (with butter, cheese, avocado, eggs, etc.) is consumed by 17.3% of students. Among students who bring lunch from home and have money available (34.1%), the average amount for expenses is $700 ± 592 (p25: $400; p75: $1000) Chilean pesos, equivalent to approximately US$0.75. Almost all students report having lunch daily (96.3%), and 74.7% also have an afternoon snack. The traditional Chilean meal, ’once-comida’ (a reinforced evening snack), is consumed by 17.4% of children, while 40.5% report having dinner.

Unexpectedly consuming some sub-groups of HPF in moderate quantities (either no consumption or occasional consumption), after adjusting by sex, commune and nutritional status (BMI/age), increased the likelihood of meeting dietary guidelines: candies and sweets (OR: 0.72; p = 0.04), pies with or without filling (OR: 0.63; p = 0.007), and salted snacks (e.g., French fries, cheese-flavored puffs) (OR: 0.67; p = 0.02) (Table 4).

**Table 4:** Influence of acceptable consumption on the probability of schoolchildren (n = 1,094) meeting three or more dietary recommendations from the Chilean Dietary Guidelines in seven schools of in southern area of Santiago, Chile, 2019.

Variables	Unadjusted			Adjusted		
	OR	p	CI 95%	OR	p	CI 95%
Sandwiches, pizzas, and other items (junk food)	1.65	0.01	1.11–2.440.90	1.64	0.01	1.11–2.43
Processed juices and sugary sodas	1.13	0.61	0.70–1.84	1.14	0.59	0.70–1.86
Candies, sweets, etc.	0.73	0.04	0.53–0.99	0.72	0.04	0.53–0.98
Salted snacks (French fries, Puffs Cheese Flavored Snacks, etc)	0.67	0.02	0.45–0.93	0.67	0.02	0.48–0.93
Pies with or without filling	0.64	0.00	0.46–0.88	0.63	0.01	0.45–0.88
Nutritional status				1.02	0.85	0.79–1.32
Sex				0.92	0.61	0.69–1.24
Commune				0.97	0.57	0.89–1.06

Adjusted by sex, commune and nutritional status (BMI/age).

## Discussion

In a cohort of 1,094 schoolchildren between eight and twelve years old, studying and living in a vulnerable area of Santiago, Chile, approximately 20.0% consume the recommended portions for most of the Chilean Dietary Guidelines Based on Foods (DGBF) (water, fruits, legumes, fish, and dairy), and only 9.0% meet the recommendation for vegetables. Highly processed foods are consumed by most of the schoolchildren and the consumption of some sub-groups of HPF not in excess (in acceptable amounts) such as candies, sweets, sweet and savory biscuits (sweet snacks), salted snacks and pies with or without filling, improves the likelihood of better adherence to the DGBF. In other words, it does not interfere with the consumption of foods recommended by dietary guidelines. However, this effect was not observed for other HPF sub-groups such as sandwiches, pizzas, and other items (junk food) or processed juices and sugary sodas.

Our study demonstrates a low level of compliance with all food groups of the DGBF among students, particularly for vegetables (9.0%), and 56.6% adhere to the recommendations for the five DGBF analyzed. In Chile, only 15.0% of the population consumes the recommended five portions of fruits and vegetables per day, heavily influenced by social and economic determinants of health. The highest consumption is observed among individuals with high socioeconomic and educational levels [9]. Previous data published from the same sample of this study indicated that 25.4% of households experience moderate-to-severe food insecurity, with severe food insecurity affecting 6.4% of households. Factors such as being a migrant, low maternal education level, poor basic knowledge in nutrition, and when the father is responsible for food purchases significantly increase the risk of food insecurity [5].

Chile presents a high prevalence of obesity in schoolchildren. A recent national census of 524,274 schoolchildren (2023) indicates that the prevalence of malnutrition by excess was 50.0% and the prevalence of stunting was 2.6%. In the first-grade level (6–7 years old), the prevalence of excess weight was 49.3%, and in the fifth-grade level (9–10 years old), it was 61.5% [24]. These results require a different analytical perspective to evaluate the high health risk of inadequate food consumption among children and our results reaffirm that it is important to improve the consumption of food groups promoted by DGBF and discourage the consumption of HPF, with different emphasis in some sub-groups of HPF.

In recent years, Chile has implemented regulations aimed at modifying the food environments in schools by prohibiting the sale and advertising of packaged foods with front-of-package labels (FOPL), high in critical nutrients (calories, sugar, saturated fat) [25, 26]. The results of this regulation showed a decline in the purchase of beverages marked "High in" [27], and the industry modified the content of some nutrients (e.g., sugars) to avoid using the FOPL [28], however, a negative externality was the increased consumption of non-nutritive sweeteners [17].

Interestingly, 32.0% of the students in our study have double breakfast (at home and at school), and 43.6% bring their morning snack from home. A recent study by our group (unpublished data, 2023) demonstrated that 60.0% [29] of these snacks are packaged and “High in” critical nutrients, indicating an increased consumption of HPF during this period. Using a structured food frequency questionnaire [22], we observed a high consumption of HPF, but with differences between subgroups. The analysis was conducted considering that non-consumption of HPF is very low in schoolchildren, and for this reason, we considered an acceptable consumption when the values vary between non-consumption and consumption of one portion per day or per week, depending on the subgroup. The least adequacy was observed for the subgroup of processed juices and sugary sodas, with only 10.4% of consumption considered acceptable (1 glass or less per day), reflecting the high valuation of this kind of HPF on Chilean population [30].

A recent study highlighted the importance of considering the possible differences between subgroups of ultra-processed foods (according to the NOVA Classification) in the risk analysis of their consumption and the incidence of multimorbidity. Higher consumption was associated with an increased risk of multimorbidity, including cancer and cardiometabolic diseases, but not all subgroups of ultra-processed foods present the same risk. Animal-based products (HR: 1.09, 95% CI: 1.05–1.12) and artificially and sugar-sweetened beverages (HR: 1.09, 95% CI: 1.06–1.12) present the highest risk. However, ultra-processed breads and cereals (HR: 0.97, 95% CI: 0.94–1.00) and plant-based alternatives (HR: 0.97, 95% CI: 0.91–1.02) were not associated with an increased risk of multimorbidity [31].

In our study, the consumption of some subgroups of HPF in acceptable amounts (1 portion or less per week) was not associated with the risk of not complying the DGBF: candies and sweets (OR: 0.72; p = 0.04), sweet and savory biscuits (snacks) (OR: 0.67; p = 0.02), pies with or without filling (OR: 0.63; p = 0.007) and salted snacks (OR: 0.67; p = 0.02). However, the same direction was not observed for sandwiches, pizzas, and other junk foods (OR: 1.64; p = 0.01), or for processed juices and sugary sodas (OR: 1.14; p = 0.59). These results indicate that perhaps the consumption of these subgroups may replace the consumption of FBDG groups, displacing the intake of healthier foods. The adjusted models likely reflect more accurate associations, emphasizing the importance of controlling for confounding variables in epidemiological studies to ensure valid interpretations of the data.

In the school environment, it is important to reduce exposure to HPF by improving regulations to ban these kinds of foods in school areas, strengthening school programs, and enhancing school kiosks to increase the availability of healthy foods adapted to school consumers [32, 33]. An analysis of the food environment, around 100 meters of schools in a vulnerable area of Santiago, Chile, found a lack of healthy foods, which was related to certain socio-economic determinants and multidimensional poverty [34]. This indicates the need to regulate the sale of unhealthy foods around schools and to promote the sale of healthy foods.

This study has some limitations. The use of a semi-structured food frequency questionnaire may have constrained the classification of HPF subgroups. Despite efforts to minimize recall bias through trained nutrition professionals and portion size reference images, the reliance on self-reported dietary data in children is prone to recall and social desirability biases, potentially leading to under- or overestimation of food intake. More objective tools, such as direct observation or food diaries, could improve accuracy but are less feasible in large-scale studies like ours.

Additionally, the focus on schoolchildren from south area of Santiago, Chile, limits the generalizability of findings to other regions or socioeconomic contexts, where dietary habits and access to HPF may vary. Moreover, as a cross-sectional study, it cannot establish causality between HPF consumption and health outcomes. Unaccounted factors, such as parental influence, peer behavior, and home food availability, may also shape dietary patterns [35], emphasizing the need for future research to address these variables and investigate behavioral and environmental determinants of dietary choices.

A strength we want to highlight in this study is its detailed disaggregation of HPF into subcategories, allowing for a nuanced analysis of their specific health impacts. HPF varies significantly in nutritional quality; for instance, snacks and sugary drinks have distinct effects compared to fortified or protein-rich foods. This approach enhances the study’s relevance by providing targeted insights for public health interventions and more specific dietary recommendations. Considering the challenges of eliminating HPF from modern diets, as highlighted by Forde (2023), promoting strategies to reformulate and improve the nutritional quality of processed foods, reducing the amount of critical nutrients and added additives, could be a more practical and impactful approach to addressing health risks while ensuring accessibility and affordability in diverse populations [36]. Additionally, increasing the availability of healthier foods, particularly in school environments, is crucial.

## Conclusions

Our results contribute to reaffirm the need to enhance healthy food consumption among schoolchildren and to discourage the intake of HPF, particularly focusing on specific sub-groups of HPF that may increase the risk of displacing the consumption of food groups recommended by the DGBF. Only 20.0% of students consume the DGBF recommendations (fruits, vegetables, legumes, water, fish) and only 9.0% consumes the portions recommended for vegetables (three portions/day). Most of the students consume HPF regularly and only 5.0% do not consume this food group.

Our findings regarding the quality of schoolchildren’s diets are consistent with those reported in other countries, characterized by a high consumption of highly processed foods and a low intake of healthy foods such as fruits, vegetables, legumes, and fish. International recommendations to address this issue consistently emphasize measures such as taxing unhealthy foods, adopting front-of-package warning labels for packaged foods high in critical nutrients, regulating the advertising of such foods, and modifying food environments, particularly in schools. Over the past decades, Chile has implemented many of these regulatory measures, contributing to the establishment of healthier environments [26]. According to Oliveira et al, “individual responsibility can only have full effect when people have access to a healthy lifestyle” [37].

Addressing the issue of school obesity requires a comprehensive set of measures, including both individual and structural initiatives, in line with the socio-ecological model of intervention. Within the school environment, it is essential to implement interventions that modify the curriculum by integrating a healthy lifestyle content into subjects such as mathematics, language, and history [38]. Additionally, improving the analysis of food consumption patterns that use novel methods is crucial for identifying potential causal relationships [39].

## Supporting information

S1 File Inclusivity in global research. (DOCX)
